# Letter to “Access to rehabilitation after stroke in Brazil (AReA study): multicenter study protocol”

**DOI:** 10.1055/s-0043-1771165

**Published:** 2023-07-26

**Authors:** Vinícius Viana Abreu Montanaro

**Affiliations:** 1 Rede Sarah de Hospitais de Reabilitação, Brasília DF, Brazil.

Reply to the letter regarding the article entitled: “Access to rehabilitation after stroke in Brazil (AReA study): multicenter study protocol”

Dear editor,

Cacho et al. 1 intend to conduct a nationwide study that focuses on access to rehabilitation for patients after a stroke. This research is fundamental and necessary. However, the primary problem is that they consider physical therapy as the main outcome, stating that other types of rehabilitation (such as speech language pathologists, physiatrists, occupational therapy, etc.) are scarce and probably won't be measured in terms of the number of patients referred to rehabilitation. Consequently, the questionnaire neglects most of these aspects. A questionnaire can be an effective tool to systematically gather this information. By including all aspects of rehabilitation in the questionnaire, stroke patients can provide detailed and accurate feedback about their recovery journey, including their physical symptoms, emotional well-being, and social support. 2

Furthermore, a comprehensive questionnaire can help healthcare professionals identify the areas where a stroke patient may need additional support or resources. For example, if a patient reports difficulties with their daily activities, such as bathing or dressing, then rehabilitation professionals can provide targeted interventions to help them regain their independence. 2

Multidisciplinary care is crucial in stroke rehabilitation because it provides a comprehensive approach to addressing the complex needs of stroke patients. Stroke can have a significant impact on multiple areas of a person's life, including their physical, cognitive, emotional, and social functioning. 3 Therefore, a team of healthcare professionals with diverse expertise is needed to address all of these aspects of recovery.

By working together, a multidisciplinary team can ensure that stroke patients receive coordinated and effective care that addresses all of their needs. This can result in improved outcomes, including faster recovery, reduced disability, and a better quality of life for stroke survivors. 4 Therefore the need for those aspects to be reinforced in the study protocol

Knowing that stroke patients received multidisciplinary care can also provide reassurance to family members and caregivers, who may be concerned about their loved one's recovery. 3 It can help them feel confident that their loved one is receiving the best possible care and support during this challenging time.

Rehabilitation after stroke can involve various interventions, including physical therapy, occupational therapy, speech therapy, psychological support, and social work services. Each of these interventions can have a significant impact on a person's recovery, and neglecting any one of them can result in incomplete or inaccurate conclusions about the effectiveness of stroke rehabilitation.

Furthermore, neglecting some aspects of rehabilitation after stroke can lead to underestimating the true impact of stroke on a person's life. Stroke survivors often face significant challenges in their daily lives, including difficulty with mobility, communication, and daily living activities. 4 Neglecting these aspects of rehabilitation can result in overlooking the true impact of stroke and its effect on a person's quality of life.

In summary, including all aspects of rehabilitation in a questionnaire for stroke patients is essential to ensure that healthcare professionals have a complete understanding of a patient's recovery needs and experiences. This information can help guide the development of tailored rehabilitation programs that address a patient's individual needs and goals.
